# Effector proteins of rust fungi

**DOI:** 10.3389/fpls.2014.00416

**Published:** 2014-08-20

**Authors:** Benjamin Petre, David L. Joly, Sébastien Duplessis

**Affiliations:** ^1^INRA, UMR 1136 Interactions Arbres/Microorganismes, Centre INRA Nancy LorraineChampenoux, France; ^2^UMR 1136 Interactions Arbres/Microorganismes, Faculté des Sciences et Technologies, Université de LorraineVandoeuvre-lès-Nancy, France; ^3^The Sainsbury Laboratory, Norwich Research ParkNorwich, UK; ^4^Département de Biologie, Université de MonctonMoncton, NB, Canada

**Keywords:** Pucciniales, rust fungi, genomics, transcriptomics, effectoromics

## Abstract

Rust fungi include many species that are devastating crop pathogens. To develop resistant plants, a better understanding of rust virulence factors, or effector proteins, is needed. Thus far, only six rust effector proteins have been described: AvrP123, AvrP4, AvrL567, AvrM, RTP1, and PGTAUSPE-10-1. Although some are well established model proteins used to investigate mechanisms of immune receptor activation (avirulence activities) or entry into plant cells, how they work inside host tissues to promote fungal growth remains unknown. The genome sequences of four rust fungi (two Melampsoraceae and two Pucciniaceae) have been analyzed so far. Genome-wide analyses of these species, as well as transcriptomics performed on a broader range of rust fungi, revealed hundreds of small secreted proteins considered as rust candidate secreted effector proteins (CSEPs). The rust community now needs high-throughput approaches (effectoromics) to accelerate effector discovery/characterization and to better understand how they function *in planta*. However, this task is challenging due to the non-amenability of rust pathosystems (obligate biotrophs infecting crop plants) to traditional molecular genetic approaches mainly due to difficulties in culturing these species *in vitro*. The use of heterologous approaches should be promoted in the future.

## THE KNOWN RUST FUNGAL EFFECTOR PROTEINS

Plant pathogens secrete effector proteins into host tissues to promote infection through the manipulation of host processes (Win et al., 2012). During host colonization, rust fungi form haustoria that invaginate the host plasma membrane within the host cell cavity. These structures mediate the molecular traffic between the parasite and its host, and notably the delivery of effector proteins into host cells (Rafiqi et al., 2012), although other structures such as infection hyphae are also likely to be involved in this molecular traffic (Rafiqi et al., 2010). Until now, six effector proteins have been identified in three different rust species: AvrM, AvrL567, AvrP123, and AvrP4 in the flax rust fungus *Melampsora lini*, the Rust Transferred Protein RTP1 in the bean rust fungus *Uromyces fabae*, and PGTAUSPE-10-1 in the wheat stem rust fungus *Puccinia graminis* f. sp. *tritici* (**Table 1**; Kemen et al., 2005; Ellis et al., 2007; Upadhyaya et al., 2014). They are all secreted proteins expressed in haustoria, with no clearly identified biochemical function. How they promote fungal growth inside host tissues remains unknown (**Table 1**). In contrast, their avirulence (Avr) properties (i.e., the ability to trigger specific immune responses) and/or their trafficking mechanisms (i.e., how they enter plant cells) are better understood.

**Table 1:** Rust effector proteins.

Effector protein	aa residues (mature)	Signal peptide	Expression	Localization in infected tissues	Avr property (immune receptor)	Biochemical function	Role in virulence
AvrM	284–347	Yes	Haustorium^a^	Haustorium, EHMx, plant cytosol^a^	Yes (M)	nd	nd
AvrL567	127	Yes	Haustorium	Plant cytosol	Yes (L5, L6, L7)	nd	nd
AvrP123	94	Yes	Haustorium	Plant nucleus	Yes (P, P1, P2, P3)	nd	nd
AvrP4	65	Yes	Haustorium	Plant cytosol	Yes (P4)	nd	nd
RTP1	201	Yes	Haustorium^a^	Haustorium/EHMx/plant cytosol/ plant nucleus^a^	nd	Protease inhibitor/filament-forming	nd
PGTAUSPE-10-1	np	np	Haustorium	nd	yes^b^	nd	nd

*The table details the rust fungi effector proteins reported so far.*

*Avr, Avirulence; aa, amino acid; EHMx, extra-haustorial matrix; nd, not determined; ND, not detected; np, not published.*

a Direct evidence of the presence of the protein acquired by immunolocalization.

b *a host-specific toxic effect was detected*.

The four *M. lini* effector proteins were first identified as effectors due to their Avr properties (Ellis et al., 2007). More recently, a screen with a bacterial protein delivery system in wheat revealed the *P. graminis* f. sp. *tritici* protein PGTAUSPE-10-1 which causes cell death in the host line carrying the resistance gene Sr22; PGTAUSPE-10-1 was thus considered as a candidate AvrRs22 effector (Upadhyaya et al., 2014). *M. lini* AvrL567 and AvrM are model Avrs for the study of effector recognition by immune receptors. Both proteins are recognized inside plant cells by specific immune receptors following a direct physical interaction (**Table 1**; Dodds et al., 2004, 2006; Catanzariti et al., 2006, 2010). For both effectors, 3D structure-driven amino acid substitutions revealed multiple contact points mediating the interaction with their cognate receptor (Wang et al., 2007; Ravensdale et al., 2011; Ve et al., 2013). Amino acid residues within these contact points are highly variable, suggesting that an arms race is taking place between these effectors and their corresponding receptors. Such knowledge of Avr-receptor interactions is valuable for engineering improved immune receptors with expanded effector recognition (Harris et al., 2013; Segretin et al., 2014), which may ultimately help to develop broad-spectrum resistance in plants (Dangl et al., 2013).

All six rust effector proteins are thought to be translocated from haustoria into host cells (**Table 1**). RTP1 and AvrM have been directly shown to traffic from haustoria to plant cells during infection (Kemen et al., 2005, 2013; Rafiqi et al., 2010), whereas the direct recognition of AvrM and AvrL567 by cytosolic plant immune receptors indirectly demonstrates their internalization in the plant cell (Ellis et al., 2007). Current mechanistic models based on pathogen-free assays suggest that AvrP4, AvrM, and AvrL567 proteins can enter plant cells autonomously (Catanzariti et al., 2006; Kale et al., 2010; Rafiqi et al., 2010). Rafiqi et al. (2010) further showed that AvrL567 and AvrM cell entry is mediated by divergent N-terminal uptake domains, carrying hydrophobic residues that are critical for cell entry in the case of AvrM (Ve et al., 2013). This model and the assays used to build it are currently debated, and the need to study effector trafficking during the infection has been stressed (Petre and Kamoun, 2014).

Effector proteins are anticipated to be key molecules for pathogenicity, although very little is known about how they function within host tissues. Among the six characterized rust effectors, none possess a clearly identified biochemical function or a detected virulence activity (**Table 1**). Indeed, *M. lini* transgenic lines silencing AvrL567 did not show any reduced growth on flax, suggesting that this effector is not required for full virulence (Lawrence et al., 2010). As discussed by the authors, this could be explained by a high functional redundancy in the *M. lini* effector repertoire (Lawrence et al., 2010). Such redundancy was also observed in the effector repertoires of bacterial plant pathogens (Kvitko et al., 2009), and represents an obstacle for the functional characterization of virulence effector functions through genetic approaches. However, recent progresses have been made regarding RTP1, a conserved rust effector that seems to work as a protease inhibitor (Pretsch et al., 2013). On the other hand, Kemen et al. (2013) reported that RTP1 accumulates within the host-parasite interface and forms filaments. The authors proposed a role as a structural effector, possibly stabilizing fungal structures during infection. A model that integrates the different RTP1 localizations and proposed functions remains to be drawn. Several methods for the genetic transformation of *M. lini* and *U. fabae*, as well as for host-induced gene silencing (HIGS) of *Puccinia triticina* have been reported (Lawrence et al., 2010; Djulic et al., 2011; Panwar et al., 2013). Such methods, although they are still at various stages of development, represent valuable tools to investigate the contribution of individual effectors to virulence during infection.

## POST-GENOMIC APPROACHES IDENTIFY A PLETHORA OF RUST SECRETED PROTEINS CONSIDERED AS CANDIDATE EFFECTORS

In the past few years, a typical profile has emerged for plant pathogen effectors. Fungal proteins are usually considered candidate secreted effector proteins (CSEPs) if they possess a signal peptide for secretion, a small size and no other targeting sequence or transmembrane domains (Stergiopoulos and de Wit, 2009; Rouxel and Tyler, 2012; Saunders et al., 2012). Such CSEPs attract more attention when they are expressed during infection or when they present signatures of rapid evolution. Besides, expression in specific infection structures such as haustoria, often considered as a major site of effector delivery, provides another level of information. Some authors also take advantage of conserved amino acid motifs or predicted protein structures to establish large CSEP classes (Godfrey et al., 2010; Pedersen et al., 2012). Homology to known rust effectors and organization in gene families or in physical clusters have also been considered to refine these sets of CSEPs (Hacquard et al., 2012; Saunders et al., 2012). In rust fungi, such criteria have been applied in the frame of effector mining pipelines that combined genome-wide analyses and transcriptomics to reveal amazingly rich catalogs of rust CSEPs (Cantu et al., 2011, 2013; Duplessis et al., 2011a; Fernandez et al., 2012; Hacquard et al., 2012; Saunders et al., 2012; Garnica et al., 2013; Zheng et al., 2013; Bruce et al., 2014; Link et al., 2014; Nemri et al., 2014; **Table 2**).

**Table 2:** Secreted proteins considered as rust effector candidates in transcriptome studies.

Species	Interaction, biological stage	Transcriptome approach	Number of transcripts detected	Detailed analysis of CSEPs	Publication
*Hemileia vastatrix*	Infected leaves	454-pyrosequencing GS-FLX titanium	6,763 fungal transcripts	382 predicted CSEPs	Fernandez et al. (2012)
*H. vastatrix*	Urediniospores and appressoria	454-pyrosequencing GS-FLX titanium	9,234 unique fungal transcripts	516 predicted CSEPs; abondant among the most highly expressed genes, particularly *in planta*	Talhinhas et al. (2014)
*Melampsora larici-populina*	Laser capture microdissection of infected leaves	Custom whole-genome oligoarrays	7,288 to 8,145 transcripts expressed in uredinia or in mesophyll tissues	19 CSEPs in the 25 most highly up-regulated transcripts in palisade mesophyll (haustoria) compared to uredinia	Hacquard et al. (2010)
*M. larici-populina*	Infected leaves, urediniospores	Custom whole-genome oligoarrays	>7,500 transcripts expressed in each biological condition tested	509 of 1,184 predicted CSEP genes expressed* in planta*; 50 CSEP among the top 100 genes up-regulated *in planta*	Duplessis et al. (2011a)
*M. larici-populina*	Time-course infection of leaves	Custom whole-genome oligoarrays	<500 early expressed transcripts; up to 8 326 transcripts *in planta*	270 CSEP genes specifically expressed *in planta*; distinct sets of >500 CSEP genes coordinately expressed along the time course	Duplessis et al. (2011b)
*M. larici-populina*	Early infected leaves	454-pyrosequencing GS-FLX titanium	90,398 contigs; 649 reads aligned to 361 fungal genes	19 early expressed CSEP genes among 40 fungal genes supported by more than 3 reads	Petre et al. (2012)
*M. larici-populina*	Telia (autumn)	Custom whole-genome oligoarrays	9,588 transcripts expressed in telia	11 SSP genes specifically expressed in telia; 113 SSP genes up-regulated in telia *vs.* uredinia	Hacquard et al. (2013)
*Phakopsora pachyrhizi*	Purified haustoria	454-pyrosequencing GS-FLX titanium	4,483 *P. pachyrhizi* unique contigs	156 contigs encoding CSEPs	Link et al. (2014)
*P. pachyrhizi*	Infected leaves	Illumina GA II	32,940 *P. pachyrhizi* contigs	176 predicted CSEP genes	Tremblay et al. (2012)
*P. pachyrhizi*	Time-course infection of leaves	Illumina GA II	Up to 12,284 *P. pachyrhizi* transcripts expressed	Not mentioned	Tremblay et al. (2013)
*Puccinia graminis* f. sp.*tritici*	Infected leaves, urediniospores	Custom whole-genome oligoarrays	9,818 transcripts expressed in total	442 of 1,106 predicted CSEP genes expressed* in planta*; 29 CSEPs in top-100 *in planta* up-regulated genes	Duplessis et al. (2011a)
*Puccinia striiformis* f. sp.* tritici* (5 isolates)	Infected leaves and purified haustoria	Illumina Genome Analyzer II	12–28.8 Millions reads from infected leaves and purified haustoria	933 CSEPs; 57 and 31 CSEP genes induced or repressed in haustoria *vs. in planta*, respectively	Cantu et al. (2013)
*P. striiformis* f. sp.* tritici*	Purified haustoria and urediniospores	454-pyrosequencing GS-FLX titanium and Illumina GA II	12,282 transcripts from combined transcriptomes	437 Haustoria Secreted Proteins (HSP); expression confirmed for 71 HSP genes by RT-qPCR	Garnica et al. (2013)
*Puccinia triticina* (6 isolates)	Infected leaves	Illumina RNA-Seq	222,571 fungal reads	543 CSEP transcripts (445 shared by the 6 isolates)	Bruce et al. (2014)
*Uromyces appendiculatus*	Purified haustoria	454-pyrosequencing GS-FLX Titanium	7,582 *U. appendiculatus* contigs	413 contigs encoding CSEPs	Link et al. (2014)

This table compiles the most recent genome-scale transcriptome studies in rust fungi (i.e., custom genome oligarrays and 454/Illumina-based RNA-Seq). Identification of expressed CSEPs is detailed. See Duplessis et al. (2012) for a detailed analysis of previous transcriptome studies in rust fungi based on Sanger expressed sequence tags or cDNA-arrays.

### GENOME-WIDE ANALYSES OF CSEPs

The genome sequences of four rust species have been published so far: *Melampsora larici-populina* (poplar leaf rust fungus; Duplessis et al., 2011a), *M. lini* (flax rust fungus; Nemri et al., 2014), *P. graminis* f. sp. *tritici* (wheat stem rust fungus; Duplessis et al., 2011a) and *Puccinia striiformis* f. sp. *tritici* (wheat stripe rust fungus; Cantu et al., 2011, 2013; Zheng et al., 2013). Genome-wide effector mining in these four species revealed hundreds of genes encoding CSEPs. In *M. larici-populina*, 1,184 CSEPs have been identified from 1,898 genes encoding predicted secreted proteins (Duplessis et al., 2011a). In *M. lini*, 762 priority CSEPs were selected from 1,085 genes encoding predicted secreted proteins (Nemri et al., 2014). In *P. graminis* f. sp. *tritici*, 1,106 CSEP genes were selected from 1,934 genes encoding predicted secreted proteins (Duplessis et al., 2011a). In *P. striiformis* f. sp. *tritici*, different reports of selected sets of CSEPs have been published. In this rust fungus, a total of 2,092 CSEP coding genes were considered in isolate CY-32 (Zheng et al., 2013) while the draft genome of isolate PST-130 led to 1,088 filtered CSEPs out of 1,188 genes coding predicted secreted protein (Cantu et al., 2011). However, genome re-sequencing of four other isolates and cross-comparison with PST-130 has led to a revision of gene numbers and to a larger set of 2,999 predicted CSEPs (Cantu et al., 2013).

All rust fungi genomes are marked by expansions of gene families, particularly those encoding secreted proteins. For instance, the largest CSEP gene family in *M. larici-populina* includes 111 members (Duplessis et al., 2011a). Noteworthy, a part of these genes were not predicted by algorithms but rather found by manual curation, highlighting the importance of expert annotation of these atypical gene families of small proteins (Duplessis et al., 2011a; Hacquard et al., 2012). This last observation is important to consider when performing cross-comparison between genomes showing different degrees of annotation. Since RXLR or LXLFLAK conserved motifs found in oomycetes helped defining large effector families (Win et al., 2007), a particular focus on motif search was given in rust CSEPs. The motif [YFW]xC has been reported in the genomes of obligate biotrophic pathogens of cereals, including *P. graminis* f. sp. *tritici* (Godfrey et al., 2010). In *M. larici-populina*, this motif is common, eventually with positional constraints, but with no restriction to the N-terminus of CSEPs (Hacquard et al., 2012). Nonetheless, functional and structural characterization for the [YFW]xC motif is lacking at the moment, and no evidence for a role in translocation has been provided so far.

Another common trend observed in rust candidate effector repertoires is the large proportion of species-, family- or order-specific CSEPs (Duplessis et al., 2014a). A large majority of species-specific CSEP genes (nearly 70%) were first observed in *M. larici-populina*. With the sequencing of the flax rust genome this number has reduced, as only 4% of the *M. lini* CSEP genes were found to be species-specific and more than half had a homolog in one of the three other sequenced rust genomes (Nemri et al., 2014). Interestingly, *M. lini Avr* genes homologs are only found in *M. larici-populina* and thus could be considered family-specific effectors, whereas other genes such as *Uromyces* spp.* RTP1* or some Haustorially Expressed Secreted Proteins (HESPs) identified in *M. lini* are conserved across rust fungi (Fernandez et al., 2012). Sequencing more genomes among Pucciniales, particularly in uncovered taxonomic families, will definitely help defining the common set of core rust effectors and those that may be related to host adaptation (Duplessis et al., 2014b).

### TRANSCRIPTOMICS IDENTIFY CSEPs IN MANY RUST SPECIES

Rust fungi have rather large genomes (89–190 Mb) and an important content in repetitive elements (>43% of total genomes), which impedes the systematic sequencing and assembly of targeted species (Duplessis et al., 2014b). Indeed, genome size estimates for certain rust species go beyond the numbers given above (Leonard and Szabo, 2005; Tavares et al., 2014). Whole-genome oligoarrays or RNA-Seq has thus proven to be useful in gathering relevant information about the transcriptomes of rust fungi. A strong stage specific regulation of protein secretion has been demonstrated in *U. fabae* (Link and Voegele, 2008), and novel high-throughput approaches confirmed a coordinated expression of CSEPs during host infection, in a temporal (expression at specific time-points) or spatial (expression in specific structures) manner (**Table 2**). For instance, transcripts profiling during time-course infection of poplar leaves by *M. larici-populina* revealed waves of expression for more than 500 CSEP transcripts (Hacquard et al., 2010; Duplessis et al., 2011b; Petre et al., 2012). Moreover, such temporal succession of expression patterns has been confirmed in other rust species such as *Hemileia vastatrix* (Fernandez et al., 2012), *P. striiformis* f. sp. *tritici* (Cantu et al., 2013), and *Puccinia triticina* (Bruce et al., 2014). This highlights the need for a better understanding of expression regulation in rust fungi, whether by transcription factors or via epigenetic control, such as reported in *Phytophthora infestans* or in *Leptosphaeria maculans* (Judelson, 2012; Soyer et al., 2014).

Interestingly, different reports showed that *U. fabae* RTP1 homologs may have different localizations (Kemen et al., 2005; Hacquard et al., 2012). RTP1 also exhibits a dynamic pattern of localization in the extra-haustorial matrix and within host cells during the infection process (Kemen et al., 2013), illustrating once more that rust effectors deployment is probably finely regulated in time and space. In this regard, a major issue with *in planta* expression study is the occurrence of different fungal cell types (germ tubes, appressoria, substomatal vesicles, infection hyphae, haustoria, sporogenous hyphae, and newly formed spores), which implies that the observed expression levels are often a mixture of different cell types at different stages. After the seminal paper that described a method to purify haustoria from the bean rust fungus (Hahn and Mendgen, 1997) and the one reporting on *M. lini* HESPs that included several Avr genes (Catanzariti et al., 2006), haustoria purification has been combined with RNA-Seq studies to prioritize CSEPs likely delivered by these infection structures (Cantu et al., 2013; Garnica et al., 2013, Link et al., 2014). Laser capture microdissection has also been coupled to transcriptomics to distinguish between biotrophic and sporogenous areas in poplar leaves infected by *M. larici-populina* (Hacquard et al., 2010). This study demonstrated that CSEPs are predominantly and highly expressed in the area containing infection hyphae and haustoria.

In order to complete their life cycle, heterecious rust fungi infect two unrelated host species. To do so, it is likely that they express host-specific effector sets. However, except for the wheat leaf rust *P. triticina* (Xu et al., 2011), only a small portion of the life cycle has been surveyed in most rust species. Recently, in order to expand our understanding of the transcriptome of *M. larici-populina*, gene expression analyses were conducted on rust telia collected from decaying leaves (Hacquard et al., 2013). This study revealed that CSEP-encoding genes were expressed in these tissues, suggesting that CSEPs might have additional roles unrelated to the interaction with the living host plant (Hacquard et al., 2013). Ongoing transcriptome profiling studies in different rust species will help to determine the sets of CSEP genes expressed along the life cycle. Such studies may reveal CSEPs with a host-specific expression, which represent host-adapted effectors (Duplessis et al., 2014b).

## TOWARDS UNIFIED EFFECTOR MINING AND EFFECTOROMICS PIPELINES

Various studies combined genome sequencing and transcriptomics to provide sets of CSEPs. Automated pipelines for effector mining should be unified and systematically applied to forthcoming rust fungi genomes to provide a solid foundation for future comparative analyses in Pucciniales. However, an important point to consider is the need for an accurate curation of CSEP-encoding genes in these genomes and the screening of additional time points in time-course studies and/or spore stages. Some early genome-wide surveys of CSEPs in plant interacting fungi arbitrarily focused on small proteins because of the commonly observed small size of effectors and in order to reduce manual gene curation efforts (Stergiopoulos and de Wit, 2009, Duplessis et al., 2011a). Considering that rust fungi effectors can exhibit greater size (e.g., *M. lini* AvrM), such an arbitrary cut-off should not be considered in future analyses of rust CSEPs.

To face the growing number of CSEPs made available by effector mining studies, and to better understand their functions in plant cells, we need tools to study them directly *in planta*. This relies on the ability to genetically transform the plant to perform high-throughput functional analyses (also referred to as “effectoromics”). Rust fungi hosts (e.g., wheat, soybean, flax, or poplar), are not easily amenable to molecular genetic approaches. However, non-host model plants can be used to characterize and screen CSEPs. For instance, the *Agrobacterium*-mediated transient genetic transformation of *Nicotiana benthamiana* has proven useful to rapidly express effector proteins into plant cell, but has been largely ignored in rust effector biology. This system allows combining many different approaches (cell-biology, protein biochemistry, hypersensitive response and infection assays) all in one. Thus, such approaches may help in (1) determining the sub-cellular localization of candidate effector proteins using effector-fluorescent protein fusions, (2) identifying interacting partners within protein complexes, (3) detecting candidate effector capacity to enhance susceptibility during infection with selected *N. benthamiana* pathogens (thus validating a role in virulence), and (4) testing their recognition by specific immune receptors.

## AUTHOR CONTRIBUTIONS

Benjamin Petre and Sébastien Duplessis compiled data from the literature and drafted the manuscript. All the authors wrote and revised the article.

## Conflict of Interest Statement

The authors declare that the research was conducted in the absence of any commercial or financial relationships that could be construed as a potential conflict of interest.
